# Kidney segmentation from DCE-MRI converging level set methods, fuzzy clustering and Markov random field modeling

**DOI:** 10.1038/s41598-022-23408-1

**Published:** 2022-11-05

**Authors:** Moumen El-Melegy, Rasha Kamel, Mohamed Abou El-Ghar, Mohamed Shehata, Fahmi Khalifa, Ayman El-Baz

**Affiliations:** 1 Electrical Engineering Department, Assiut University, Assiut, Egypt; 2 Computer Science Department, Assiut University, Assiut, Egypt; 3 Radiology Department, Urology and Nephrology Center, Mansoura University, Mansoura, Egypt; 4 Bioengineering Department, University of Louisville, Louisville, KY USA; 5 Electronics and Communications Engineering Department, Mansoura University, Mansoura, Egypt

**Keywords:** Biomedical engineering, Electrical and electronic engineering, Radiography

## Abstract

Early diagnosis of transplanted kidney function requires precise Kidney segmentation from Dynamic Contrast-Enhanced Magnetic Resonance Imaging images as a preliminary step. In this regard, this paper aims to propose an automated and accurate DCE-MRI kidney segmentation method integrating fuzzy c-means (FCM) clustering and Markov random field modeling into a level set formulation. The fuzzy memberships, kidney’s shape prior model, and spatial interactions modeled using a second-order MRF guide the LS contour evolution towards the target kidney. Several experiments on real medical data of 45 subjects have shown that the proposed method can achieve high and consistent segmentation accuracy regardless of where the LS contour was initialized. It achieves an accuracy of 0.956 ± 0.019 in Dice similarity coefficient (DSC) and 1.15 ± 1.46 in 95% percentile of Hausdorff distance (HD95). Our quantitative comparisons confirm the superiority of the proposed method over several LS methods with an average improvement of more than 0.63 in terms of HD95. It also offers HD95 improvements of 9.62 and 3.94 over two deep neural networks based on the U-Net model. The accuracy improvements are experimentally found to be more profound on low-contrast images as well as DCE-MRI images with high noise levels.

## Introduction

**Figure 1:**
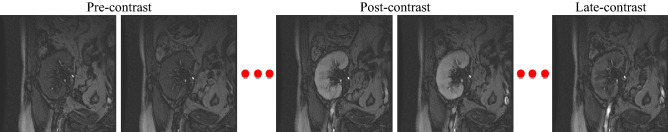
A sequence of DCE-MRI time-point kidney images for one subject manifesting the contrast change caused during the contrast agent perfusion into the kidney.

Acute rejection is the most common cause of the failure of kidney transplantation and has to be early detected to rescue the transplanted kidney^1^. Towards that end, one of the often used methods is DCE-MRI. DCE-MRI is obtained by injecting the patient with a contrast agent and capturing fast and frequent images for the kidney. Therefore, each patient typically has a dataset that contains a sequence of about 80 varying-contrast kidney images that are captured during the contrast agent perfusion^1^ as shown in Fig. 1. From these images, accurate segmentation of the kidney becomes a necessary and first step for downstream processing operations to determine the kidney status. However, it remains a challenging problem^1,2^ due to the low spatial resolution and contrast variation of the quickly-acquired images and motion resulting from patient breathing and movement.

### Related work

Over the years, extensive work has been done to figure out DCE-MRI kidney segmentation problem. Among these methods, the level set (LS) methods have been the most popular^3–9^. For example, the LS method^3^ is applied for segmenting kidneys from DCE-MRI images and the contour evolution is controlled by shape and gray-level information. However, relying only on intensity and shape information may result in incorrect segmentation results on noisy and low-contrast images. The authors^4,5^ pointed to this shortcoming and improved it by adding a 2nd-order Markov-Gibbs random field (MGRF) spatial interaction model. Khalifa et al.^6^ enhanced their earlier work^5^ via increasing the order of the MGRF model to four. To overcome the similarity between the pixel intensities in the kidney and surrounding background, Liu et al.^7^ proposed to remove the gray-level distribution from the speed function presented by Khalifa et al.^6^ and use a 5th-order MGRF spatial interaction model. To address the low contrast problem of kidney images, Al-Shamasneh et al.^8^ developed a contrast enhancement method by employing a local fractional entropy model. While some researchers did not make use of the kidney shape information (e.g., Al-Shamasneh et al.^9^), many have found this information useful^5–7,10^. Incorporating this shape information typically requires a separate registration step^5–7^ to align an input DCE-MRI image to the pre-constructed shape model in order to compensate for the patient’s motion caused during data acquisition. However, Hodneland et al.^10^ attempted to segment kidneys from DCE-MRI by executing the registration and segmentation processes simultaneously. In the LS methods, the contour evolution is guided by deriving a partial differential equation. This equation typically contains weighting parameters that need to be tuned. In contrast, Eltanboly et al.^11^ introduced a LS segmentation method that incorporates the shape and gray-level intensity information without using weighting parameters.

More recently, kidney segmentation using convolutional neural networks (CNNs) has become an area of increased research. Extensive work has been done for kidney segmentation from CT images using deep neural networks. However, the number of studies attempted to perform kidney segmentation in MRI^12–19^ is rather limited. Lundervold et al.^12^ developed CNNs-based kidney segmentation from 3D DCE-MRI employing a transfer learning approach from a pre-trained network. While Haghighi et al.^13^ presented a method based on the deep U-Net^14^ architecture, Milecki et al.^15^ developed their own 3D CNN architecture. In^16^, the authors combined two different CNNs-based approaches for segmenting kidneys from MRI images. Brunetti et al.^17^ employed a mono-objective genetic algorithm with CNNs to achieve accurate kidney segmentation from MRI data. Isensee et al.^18^ performed a multi-organ segmentation task including left and right kidneys from CT-MRI scans in CHAOS challenge dataset^19^ employing nnU-Net model. Supplementary Table S1 online summarizes the key aspects of these research efforts.

### Research gap

Lacking a sufficiently large number of annotated training data, the existing deep network methods could not achieve high segmentation accuracy. On the other hand, the aforementioned LS methods can achieve high performance in segmenting the kidney from its surroundings. However, the majority of these methods require an accurate delineation for the initial contour which was performed manually by the user. Lack of good initialization may result in the method’s complete failure or a deficit in the resulting accuracy. To overcome this problem, we have presented a fast and automated FCMLS DCE-MRI kidney segmentation method^20^ based on the principle of fuzzy c-means (FCM) clustering algorithm and LS method integration. The LS contour evolution is constrained by kidney’s shape prior information and fuzzy memberships in the kidney class. Moreover, the LS method employs a smeared-out Heaviside function making the method accurate and robust against contour initialization. Although the FCMLS method has succeeded in segmenting kidneys from DCE-MRI images, its segmentation performance is rather lower on low-contrast images, such as the ones in the pre-contrast and late-contrast parts of the time sequence in Fig. 1. In addition, the fuzzy membership values of the pixels are computed before the LS contour starts evolving and this might be not very accurate in some cases. Moreover, adopting the 1st-order shape prior method in the representation of the shape information is not accurate in some cases where some kidney pixels are not observed at all in the images used to construct the shape model.

In this paper, to overcome the aforementioned limitations of our method^20^ and to further improve its performance, we propose a new automated and robust DCE-MRI kidney segmentation method based on converging ideas from FCM clustering^21^, LS method^22^, and MRF modeling^23^. The FCM clustering algorithm and LS method have proved their efficiency in image segmentation. However, their segmentation performance drops with noisy and low-contrast images. This is due to their assumption that the pixels in object and background regions are totally separated from each other when computing the energy function. To get around this problem, statistical segmentation schemes, like MRF model, can be used. According to the MRF model, the energy function of every pixel in the image is identified by the pixel and its neighbors. Thus, using the MRF model in building the energy function enhances the segmentation accuracy and makes the segmentation robust against noise.

The main contributions of this paper can be summarized as follows. First, the FCM clustering algorithm is embedded into a LS method formulation. Thus, the membership values of the pixels are computed and updated during the LS contour evolution. Second, a 2nd-order MGRF model is incorporated into the energy function guiding the LS evolution. Third, the kidney’s shape prior model is more accurately computed using a Bayesian parameter estimation method^24^, which accounts for kidney pixels not observed during model building. The segmentation performance of the proposed method, named FML, is tested on 45 patients’ datasets and the accuracy is evaluated by DSC and HD95 metrics^2^. Experimental results verify the *consistent high* performance of the FML method on noisy, high and low-contrast images without any tuning for the weighting parameters. Another important advantage of the FML method is its ability to achieve high segmentation accuracy with randomly initialized LS contour. In order to further prove the efficiency of the FML method, we compare its segmentation accuracy with several state-of-the-art LS methods and two deep neural networks based on the U-Net model. The comparison results confirm the superior performance of the proposed FML method for kidney segmentation from DCE-MRI.

## Materials and methods

This section presents the proposed FML method which embeds the FCM clustering algorithm and MRF modeling into a LS method to achieve accurate segmentations from DCE-MRI images, taking into account prior information about kidney’s shape.

### Materials

DCE-MRI data is collected from 45 patients using a 1.5 T MRI scanner with a phased-array torso surface coil at Mansoura University Hospital, Egypt. All methods were performed in accordance with the relevant guidelines and regulations, and the patients’ informed consent was obtained. Our experiments were reviewed and approved by the institutional review board (IRB) at the University of Louisville. Each patient has a dataset of about 80 repeated temporal frames of size 256 $\times$ 256 pixels in DICOM format that were obtained via injecting the patient by a dose of 0.2 ml/kgBW of Gd-DT PA contrast agent with the rate of 3–4 ml/s. The dataset contains a sequence of varying-contrast kidney images that were acquired quickly and repeatedly at 3 s intervals during the contrast agent perfusion into the kidney. A sample sequence for one subject is shown in Fig. 1. Each image was *manually* segmented by expert radiologists at the hospital using the Adobe Photoshop software.

### Problem statement and basic notations

As already mentioned, each patient typically has a time sequence of varying contrast images of the kidney. The target is to segment kidney from each image separately. Let $I = \left\{ {I_{x,y} , \left( {x,y} \right) \in {\Omega }} \right\}$ be the intensity information of a DCE-MRI grayscale image that needs to be segmented; where $I_{x,y}$ is the intensity of the pixel $\left( {x,y} \right)$ in the image domain $\Omega$. The target is to label each pixel $\left( {x,y} \right)$ in the image as kidney ($K$) or background ($B$). The label information is represented in $L = \left\{ {L_{x,y} , \left( {x,y} \right) \in {\Omega }, L_{x,y} \in \left\{ {K,B} \right\}} \right\}$.

### Level set segmentation method with fuzzy clustering and Markov random field modeling

For segmenting the DCE-MRI input image, the LS contour $\partial \Omega$ divides the image domain $\Omega$ into kidney region $\Omega^{K}$ and background region ${\Omega }^{B}$. The LS function $\phi$ is defined as a distance map of the signed minimum Euclidean distances from every pixel $\left( {x, y} \right)$ to the contour. The distance is zero for the pixels on the contour ($\phi_{x,y} = 0$), positive in kidney region ($\phi_{x,y} > 0$), and negative in background region ($\phi_{x,y} < 0$), as illustrated in Supplementary Fig. S1 online. The LS contour is evolved to the target kidney through minimizing the energy functional of the following form:

1 $$E\left( \phi \right) = \lambda_{1} {\mathcal{L}}\left( \phi \right) + \lambda_{2} E_{{{\mathcal{F}\mathcal{C}\mathcal{M}}}} \left( \phi \right) + \lambda_{3} E_{{{\mathcal{M}\mathcal{R}\mathcal{F}}}} \left( \phi \right)$$

where $\lambda_{i} > 0$ are normalizing parameters that control the impact of the energy terms. The first term in the energy functional in Eq. (1) represents internal energy that controls the smoothness of the LS contour. The last two terms are external energy computed from the input image to attract the contour towards the kidney position. More specifically, the first term is used to compute the length of the zero LS contour and defined as:

2 $${\mathcal{L}}\left( \phi \right) = \mathop \smallint \limits_{{\Omega }}^{ } \delta_{\varepsilon } \left( {\phi_{x,y} } \right) \left| {\nabla \phi_{x,y} } \right| dx\, dy$$

where $\delta_{\varepsilon } \left( {\phi_{x,y} } \right)$ is the Dirac delta function which is the derivative of the smeared-out Heaviside function $H_{\varepsilon } \left( {\phi_{x,y} } \right)$, where both are defined as

3 $$\begin{aligned} H_{\varepsilon } \left( {\phi_{x,y} } \right) = & \left\{ {\begin{array}{*{20}l} 1 \hfill & {\phi > \varepsilon } \hfill \\ {\frac{1}{2} + \frac{\phi }{2\varepsilon } + \frac{1}{{2{\uppi }}}\sin \left( {\frac{{{\uppi }\phi }}{\varepsilon }} \right)} \hfill & { - \varepsilon \le \phi \le \varepsilon } \hfill \\ 0 \hfill & { \phi < - \varepsilon } \hfill \\ \end{array} } \right., \\ \delta_{\varepsilon } \left( {\phi_{x,y} } \right) = & \left\{ {\begin{array}{*{20}l} 0 \hfill & {\left| { \phi } \right| > \varepsilon } \hfill \\ {\frac{1}{2\varepsilon } + \frac{1}{2\varepsilon }\cos \left( {\frac{{{\uppi }\phi }}{\varepsilon }} \right)} \hfill & {\left| { \phi } \right| \le \varepsilon } \hfill \\ \end{array} } \right. \\ \end{aligned}$$

where $\varepsilon$ is a parameter that determines the width of numerical smearing. Using the smeared-out Heaviside and Dirac delta functions helps obtain a global minimizer irrespective of where the initial LS contour in the image is^22^. The FCM-based energy functional in Eq. (1) depends on the shape prior information and kidney/background memberships computed by the FCM clustering algorithm, and is defined as:

4 $$E_{{{\mathcal{F}\mathcal{C}\mathcal{M}}}} \left( \phi \right) = { }\mathop \int \limits_{{\Omega }}^{ } H_{\varepsilon } \left( {\phi_{x,y} } \right){ }{\mathcal{S}}_{{B_{x,y} }} { }\mu_{{B_{x,y} }} { }dx\,{ }dy{ } + { }\mathop \int \limits_{{\Omega }}^{ } \left[ {1 - H_{\varepsilon } \left( {\phi_{x,y} } \right){ }} \right]{ }{\mathcal{S}}_{{K_{x,y} }} { }\mu_{{K_{x,y} }} { }dx\,{ }dy{ }$$

where for a pixel $\left( {x,y} \right)$ in the image, ${\mathcal{S}}_{{L_{x,y} }}$ is the pixel’s prior probability to be kidney (${\mathcal{S}}_{{K_{x,y} }}$) or background (${\mathcal{S}}_{{B_{x,y} }}$) derived from the shape prior model and $\mu_{{L_{x,y} }}$ is kidney ($\mu_{{K_{x,y} }}$) and background ($\mu_{{B_{x,y} }}$) fuzzy memberships of this pixel. Information about how the shape prior model and fuzzy memberships are computed is provided in the following sections. The MRF-based energy functional in Eq. (1) models the relationship between the pixels and their neighbors and is defined as:

5 $$E_{{{\mathcal{M}\mathcal{R}\mathcal{F}}}} \left( \phi \right) = \mathop \int \limits_{\Omega }^{~} H_{\varepsilon } \left( {\phi _{{x,y}} } \right)~{\mathcal{M}}_{{K_{{x,y}} }} dx~dy + \mathop \int \limits_{\Omega }^{~} \left[ {~1 - H_{\varepsilon } \left( {\phi _{{x,y}} } \right)~} \right]~{\mathcal{M}}_{{B_{{x,y}} }} ~dx~dy$$

where ${\mathcal{M}}_{{K_{x,y} }}$ and ${\mathcal{M}}_{{B_{x,y} }}$ are kidney and background MRF-based energy functions that are based on the framework of Bayes theorem and explained in details below. According to the calculus of variations, the minimization of the energy functional in Eq. (1) with respect to $\phi$ is given by:

6 $$\frac{\partial \phi }{{\partial t}} = - \frac{\partial E}{{\partial \phi }} = \lambda_{1} \delta_{\varepsilon } \left( \phi \right){\text{ div}}\left( {\frac{\nabla \phi }{{\left| {\nabla \phi } \right|}}} \right) + \lambda_{2} \delta_{\varepsilon } \left( \phi \right) \left[ { {\mathcal{S}}_{K} { }\mu_{K} - {\mathcal{S}}_{B} { }\mu_{B} } \right] + \lambda_{3} \delta_{\varepsilon } \left( \phi \right) \left( { {\mathcal{M}}_{B} - {\mathcal{M}}_{K} )} \right.$$

Finally, the contour is recursively evolved to the object boundary according to

7 $$\phi_{n + 1} = \phi_{n} + \tau \frac{{\partial \phi_{n} }}{\partial t}$$

where $n$ is a time step and $\tau > 0$. Below we give more details about the proposed approach, while Supplementary Algorithms S1 and S2 outline, respectively, the steps of offline shape model construction and the full segmentation method.

### FCM membership function

For an input DCE-MRI image, the FCM clustering algorithm^21^ classifies the pixels of the image into two clusters based on Euclidean distance of the pixel from the center of the least distant cluster by minimizing the following objective function^25^:

8 $$J = \mathop \sum \limits_{{\left( {x,y} \right) \in {\Omega }}}^{ } \mathop \sum \limits_{L = 1}^{2} \mu_{{L_{x,y} }}^{2} \left\| {I_{x,y} - v_{L} } \right\| ^{2}$$

where $v_{L}$ is the center of the cluster $L$, $\mu_{{L_{x,y} }} \in \left[ {0,1} \right]$ is the membership degree of the pixel $\left( {x,y} \right)$ in the cluster $L$. In our predecessor method, the FCMLS method^20^, the FCM clustering algorithm is initially used for partitioning the pixels in the input image into kidney and background clusters, and then, the LS contour starts to evolve using the obtained clusters to control the LS contour evolution. That is, the FCM clustering algorithm is only used once at the beginning of the segmentation process. In contrast in this new method, the FCM clustering algorithm is embedded in the LS method. More specifically, the proposed method starts with initial kidney and background centroid values that are used to generate initial kidney and background clusters. From the centroid values, the kidney**/**background fuzzy memberships for every pixel $\left( {x,y} \right)$ in the image are computed using:

9 $$\mu_{{L_{x,y} }} = \frac{1}{{\left( {\frac{{\left\| {I_{x,y} - v_{L} } \right\|}}{{ \left\| {I_{x,y} - v_{K} } \right\|}}} \right)^{2} + \left( {\frac{{\left\| {I_{x,y} - v_{L} } \right\|}}{{ \left\| {I_{x,y} - v_{B} } \right\|}}} \right)^{2} }}$$

where $v_{L}$ is the centroid of the kidney cluster (when $L = K$) or the background (when $L = B$). Note that $\mu_{{K_{x,y} }} + \mu_{{B_{x,y} }} = 1$. Then, the centroid values of the kidney**/**background clusters are updated as:

10 $$v_{L} = \frac{{ \mathop \sum \nolimits_{{\left( {x,y} \right) \in {\Omega }}} {\mathcal{R}}_{L} \left( \phi \right) \mu_{{L_{x,y} }}^{2} I_{x,y} }}{{\mathop \sum \nolimits_{{\left( {x,y} \right) \in {\Omega }}} {\mathcal{R}}_{L} \left( \phi \right) \mu_{{L_{x,y} }}^{2} }}$$

where ${\mathcal{R}}_{L} \left( \phi \right) = {\mathcal{R}}_{K} \left( \phi \right) = H_{\varepsilon } \left( \phi \right)$ for $L = K$, and ${\mathcal{R}}_{L} \left( \phi \right) = {\mathcal{R}}_{B} \left( \phi \right) = \left( {1 - H_{\varepsilon } \left( \phi \right)} \right)$ for $L = B$. The membership value of a pixel to a cluster indicates the degree of pixel belongingness to this cluster and depends on how far is the pixel from the cluster’s centroid. Thus, the pixels are assigned high membership values to a certain cluster as their intensities are close to the cluster’s centroid value and low otherwise. The centroid values and fuzzy memberships of the two clusters are sequentially updated in this way at every step of the LS contour evolution. Supplementary Figure S2 online shows the results of the FCM algorithm on segmenting a DCE-MRI image into two separate clusters. Clearly, FCM clustering *alone* is not sufficient to obtain an accurate segmentation.

### Kidney shape prior model

**Figure 2:**
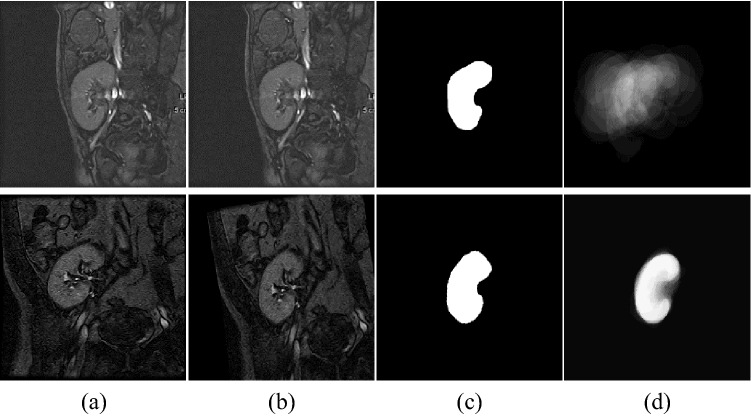
Kidney shape model construction from DCE-MRI images: (**a**) Sample images, (**b**) Images after affine transformation, (**c**) Segmented kidneys after alignment, (**d**) The shape prior model constructed using the Bayesian parameter estimation method before (top) and after (bottom) the affine registration.

As the human kidney has rather a well-defined shape, employing the shape information in the segmentation procedure will be beneficial, especially on low contrast images. To its advantage, the LS method is flexible enough to incorporate such information. The shape prior model is formed with the help of a set of DCE-MRI images gathered from different subjects. Then, kidneys are manually segmented from these images by expert radiologists. After that, the obtained binary images are registered assuming an affine transformation via mutual information maximization^26^, see Fig. 2. The shape model is eventually formed from those registered binary images.

In the previous FCMLS method, we adopt a 1st-order shape method in shape model construction, in which, the pixel’s probability to be kidney (or background) is taken as the proportion between the count that this pixel is designated as kidney (or background) and the total count of images. But, this method is not very accurate especially when a pixel $\left( {x,y} \right)$ is designated as kidney in all images. In this case, the pixel’s probability to be kidney ${\mathcal{S}}_{{K_{x,y} }}$ will be exactly 1 and ${\mathcal{S}}_{{B_{x,y} }}$ will be zero which is not very reasonable. Similarly, when this pixel is denoted as background in all images, ${\mathcal{S}}_{{B_{x,y} }}$ will be exactly 1 and ${\mathcal{S}}_{{K_{x,y} }}$ will be zero, thus hard wiring this pixel to background during evolution. In order to overcome that drawback, we use the Bayesian parameter estimation method presented by Friedman and Singer^24^ to construct the shape model.

Let ${\mathcal{N}}$ be the total number of selected images and $\ell = 2$ be the total number of possible region labels (Kidney and Background). For each pixel $\left( {x,y} \right)$ in the image, if the pixel is labelled as kidney in a group of images and background in others, its kidney/background probability is computed as follows^27^:11where ${\mathcal{O}}_{x,y}$ is the number of observed labels (equals 2 here because both labels are observed), ${\mathcal{N}}_{{L_{x,y} }} { }$ is the count of the observed label $L$ (how often this pixel is denoted as kidney or background in all images), $\beta$ is a pseudo count added to the count of each observed label, and  is a scaling factor. However, if a pixel is classified as kidney in all images, the number of observed labels ${\mathcal{O}}_{x,y}$ will be $1$, and the probability of observed label (kidney) is computed from Eq. (11). Accordingly, in this case, background is considered an unobserved label and background probability of this pixel is computed from^24^:12

Similarly, when a pixel is classified as background in all images, the number of observed labels ${\mathcal{O}}_{x,y}$ will be $1,$ and probability of observed label (background) is computed from Eq. (11) where the probability of unobserved label (kidney) is computed from Eq. (12). An example for the shape prior model constructed using the Bayesian parameter estimation method is shown in Fig. 2.

### MRF-based external energy

In order to overcome the influence of noise on the FCMLS method^20^ and to obtain more accurate segmentations, we add the MRF^23^ energy function in the energy functional. The MRF energy function constructs a square neighborhood system ${\mathbb{N}}^{w} \left( {x,y} \right)$ of size $\left( {2w + 1} \right) \times \left( {2w + 1} \right)$ for each pixel $\left( {x,y} \right)$ and finds the best segmentation label by using its neighborhood information. According to Bayes theorem, the best label segmentation can be obtained through the maximization of a posteriori (MAP) probability $P\left( {L {|} I} \right)$ defined as:

13 $$P\left( {L {|} I} \right) = \frac{{ P\left( {I {|} L} \right) P\left( L \right) }}{P\left( I \right)}$$

where $P\left( I \right)$ is constant for a segmented image and removing it will not affect the proportional relationship. $P\left( {I {|} L} \right)$ and $P\left( L \right)$ are conditional and prior segmentation probabilities that are computed for each pixel in the image using the MRF theory by taking into account its eight-neighborhoods in a 2nd-order neighborhood system, respectively. The intensities of the pixels in kidney and background regions are supposed to follow a Gaussian distribution. Thus, the conditional segmentation probability $p\left( {I {|} L} \right)$ of each pixel $\left( {x,y} \right)$ in DCE-MRI image is computed as:

14 $$p\left( {I_{x,y} {|} L_{x,y} } \right) = \mathop \prod \limits_{s \in S} \frac{1}{{\sqrt {2\pi } \sigma_{L} }}{\text{exp}}\left( { - \frac{{\left( {I_{s} - m_{L} } \right)^{2} }}{{2 \sigma_{L}^{2} }}} \right)$$

where $s$ indexes all set of pixels $S$ is in the neighborhood, ${{m}}_{L}$ and $\sigma_{L}$ are the average and standard deviation of the pixel intensities in kidney or background region and computed as:

15 $$\begin{gathered} m_{L} = \frac{{ \mathop \int \nolimits_{\Omega}^{ } {\mathcal{R}}_{L} \left( \phi \right) I_{x,y} dx \,dy }}{{\mathop \int \nolimits_{{\Omega}}^{ } {\mathcal{R}}_{L} \left( \phi \right) dx\, dy }} , \hfill \\ \sigma_{L}^{2} = \frac{{ \mathop \int \nolimits_{{\Omega}}^{ } {\mathcal{R}}_{L} \left( \phi \right) \left( {I_{x,y} - m_{L} } \right)^{2} dx\, dy}}{{\mathop \int \nolimits_{{\Omega}}^{ } {\mathcal{R}}_{L} \left( \phi \right) dx\, dy }} \hfill \\ \end{gathered}$$

where ${\mathcal{R}}_{L} \left( \phi \right) = {\mathcal{R}}_{K} \left( \phi \right) = H_{\varepsilon } \left( \phi \right)$ for $L = K$, and ${\mathcal{R}}_{L} \left( \phi \right) = {\mathcal{R}}_{B} \left( \phi \right) = \left( {1 - H_{\varepsilon } \left( \phi \right)} \right)$ for $L = B$. Similarly, the label field information $P\left( L \right)$ for each pixel is supposed to be correlated with its neighborhood. Thus, based on Hammersley-Clifford theorem^28^, the prior segmentation probability can be defined as:

16 $$P\left( {L_{x,y} } \right) = \mathop \prod \limits_{s \in S} \frac{1}{{\mathcal{T}}} exp\left( { - \mathop \sum \limits_{{{\mathcal{C} } \in {\mathbb{N}}^{w} \left( s \right)}}^{ } V_{{\mathcal{C}}} \left( {L_{s} } \right)} \right)$$

where ${\mathcal{T}}$ is a normalizing constant. $V_{{\mathcal{C}}} \left( {L_{s} } \right)$ is the clique energy function of all possible cliques ${\mathcal{C}}$ and defined as^29,30^:

17 $$V_{{\mathcal{C}}} \left( {L_{s} } \right) = \left\{ {\begin{array}{*{20}l} { - \gamma \left( {{\text{exp}}\left( { - \left| {I_{s} - I_{n\left( s \right)} } \right|} \right)} \right),} \hfill & {L_{s} = L_{n\left( s \right)} } \hfill \\ {\gamma \left( {\frac{2}{{1 + {\text{exp}}\left( { - \left| {I_{s} - I_{n\left( s \right)} } \right|} \right)}} - 1} \right),} \hfill & {L_{s} \ne L_{n\left( s \right)} } \hfill \\ \end{array} } \right.$$

where $n\left( s \right) \in {\mathbb{N}}^{w} \left( s \right)$, $\gamma$ is a constant known as the Gibbsian parameter, and $\left| {I_{s} - I_{n\left( s \right)} } \right|$ denotes the absolute difference between the intensities of center pixel and one of its eight neighbors. From the above formula of $P\left( {L_{x,y} } \right)$ in Eq. (16), it can be observed that the pixel is considered as kidney or background as most of its neighbors. Thus, the value of $P\left( {L_{x,y} } \right)$ is increased when the number of the neighborhood pixels labeled as the center pixel is increased. Moreover, the clique energy function $V_{{\mathcal{C}}} \left( {L_{s} } \right)$ is dependent on the gray-level information of pixels, which makes the FML method achieve a high-precision segmentation results. According to the theory of optimization, the kidney and background MRF energy functions are taken as:

18 $${\mathcal{M}}_{{L_{x,y} }} \approx - \log \left( { p\left( {I_{x,y} | L_{x,y} } \right) P\left( {L_{x,y} } \right) } \right) \approx \mathop \sum \limits_{s \in S}^{ } \left[ { \frac{{\left( {I_{s} - m_{L} } \right)^{2} }}{{2 \sigma_{L}^{2} }} + \log \left( {\sqrt {2\pi } \sigma_{L} {\mathcal{T}}} \right) + \mathop \sum \limits_{{{\mathcal{C}} \in {\mathbf{\mathbb{N}}}^{w} \left( s \right)}}^{ } V_{{\mathcal{C}}} \left( {L_{s} } \right) } \right]$$

## Experimental results

The proposed FML method is applied for segmenting kidneys from 45 subjects’ datasets and its efficiency is assessed using the DSC and HD95 metrics^2^. These metrics determine the similarity between the segmented kidneys and ground-truth segmentations. In the proposed method, the shape prior model is built from 30 ground-truth kidneys of different subjects; one image from each subject, using the Bayesian parameter estimation method. Several parameters are required to be set in the proposed method: the weighting parameters in Eq. (6) are experimentally chosen as ${\uplambda }_{1} = { }$ 6, ${\uplambda }_{2} = { }$ 6, and ${\uplambda }_{3} = { }$ 6. The width of numerical smearing $\varepsilon$ in Eq. (3), pseudo count $\beta$ in Eq. (11), and neighborhood size $w$ in Eq. (16) are taken 1.5, 1, and 5, respectively. The Gibbsian parameter $\gamma$ in Eq. (17) is set to 0.5. All the parameters are then fixed in all the reported series of experiments without any further tuning. We also perform several experiments to analyze the effect of the parameters on the method performance (see supplementary information online). The centroids of kidney and background clusters are initially defined as the average of pixel intensities inside and outside the initialized LS contour, respectively. All experiments are carried out in MATLAB R2015a on a pc with 1.80 GHz Intel Core i7 CPU and 16 GB of RAM.

### Comparison to other level set-based methods

We perform a comparison between the segmentation performance of the FML method and our previous FCMLS method^20^. For a fair comparison, we utilize the same images used in FCMLS method to build the shape model. We intentionally initialize the contour beyond the target kidney and close to the borders of the image in the two methods. We assess the accuracy of the two methods in segmenting kidneys from all images and particularly from low-contrast images. The total number of low-contrast images is 225 images; the first 5 time-point images from each subject dataset. Table 1 shows a quantitative comparison between the two methods in terms of mean ± standard deviation of DSC and HD95 metrics.

**Table 1:** Comparison between the segmentation accuracy of the FML and FCMLS methods.

Method	All images		Low-contrast images	
	DSC	HD95	DSC	HD95
FCMLS^20^	0.941 ± 0.042	1.78 ± 6.21	0.880 ± 0.137	8.18 ± 22.8
FML (ours)	**0.956 ± 0.019**	**1.15 ± 1.46**	**0.936 ± 0.024**	**1.94 ± 1.58**

Significant values are in [bold].

**Figure 3:**
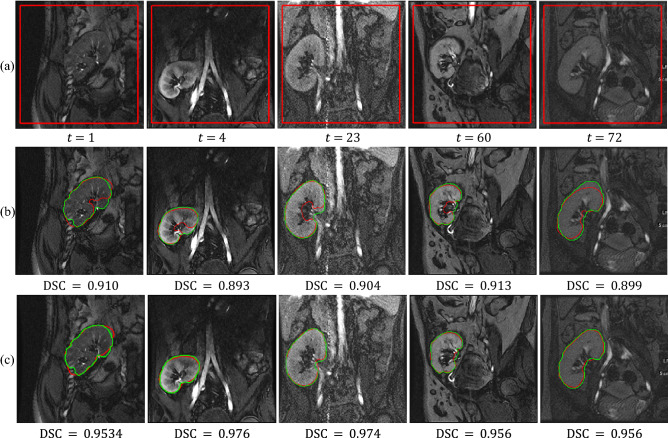
Segmentation results of FML vs. FCMLS. (**a**) DCE-MRI kidney images with initial LS contour. Segmentation results outlined in red with DSC values for the kidneys segmented by FCMLS in (**b**) and FML in (**c**). The ground-truth segmentations are superimposed on the images in green. Please refer to Supplementary Fig. S3 online for better visualization of segmentation results.

As reported in Table 1, the FML method achieves better segmentation results than the FCMLS method by providing higher mean DSC values and lower standard deviations. Moreover, the lower mean and standard deviation of HD95 values of the proposed method confirm its notable better segmentation performance than FCMLS. The standard deviation of HD95 of the FML method is significantly lower than FCMLS method which confirms that the FML method is more consistent and stable than the previous method. We can observe the significant difference between the segmentation performances of the two methods with low-contrast images. This indeed confirms that the superiority of the new FML method. Figure 3 visually shows the performance of the FML and FCMLS methods on 5 varying-contrast images of different subjects.

It can be observed from Fig. 3 that the segmentation accuracy has been notably enhanced using the FML method compared to FCMLS method. The original images are shown in the first row with an initial LS contour. The second and third rows show segmentation results of the FCMLS and FML methods, associated with the DSC values with respect to the ground-truth. The results also show that the FML method can output notably accurate segmentation results on low-contrast images compared against FCMLS method. From the time performance perspective, our comparative experiments demonstrate the faster convergence rate of the new FML method as it completes the evolution process after about 30 iterations, whereas the previous FCMLS method takes more than 40 iterations to converge. Furthermore, we compare the obtained results with the results obtained from other well-known segmentation methods, namely, the 2nd-order MGRF level-set (2nd-MGRF)^5^, shape-based (SB)^31^, vector level-set (VLS)^32^, and parametric kernel graph cut (PKGC)^33^, see Table 2.

**Table 2:** Comparison between the segmentation accuracy of the FML method and previous methods.

Method	DSC	HD95
PKGC^33^	0.820 ± 0.180	–
VLS^32^	0.902 ± 0.083	3.62 ± 7.29
SB^31^	0.912 ± 0.043	2.64 ± 1.63
2nd-MGRF^5^	0.943 ± 0.028	–
FCMLS^20^	0.941 ± 0.042	1.78 ± 6.21
FML(ours)	**0.956 ± 0.019**	**1.15 ± 1.46**

Significant values are in [bold].

The reported accuracies confirm the superior performance of the FML method over other methods, including the method^5^ which incorporates a 2nd-order MGRF model, shape prior model, and image intensity information into the LS method. This can be attributed to our better by-construction prior shape model and our well-integrated method combining all these information sources. Moreover, unlike^5^, we adopt the intensity information of pixels in computing the clique energy function. It is also important to stress that the FML method converges to accurate segmentation irrespective of the LS contour initialization, whereas the other methods require a good LS contour initialization to converge.

**Figure 4:**
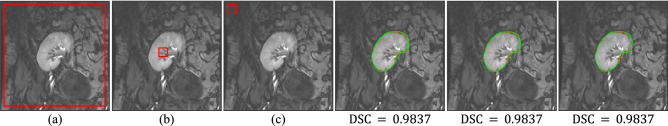
Segmentation results of the FML method on DCE-MRI image with three different LS contour initializations. (**a**–**c**) show DCE-MRI image with initial contours outlined in red. Last three columns show segmentation results of (**a**–**c**) in red while ground-truth segmentations are outlined in green, with DSC reported beneath each result.

Moreover, to verify the robustness of the FML method to contour initialization, we use it to segment kidney on a group of images with different LS initializations (see Fig. 4): outside the kidney, inside the kidney, and totally far away from the kidney. It can be seen from the results in Fig. 4 that the FML method can consistently achieve the same high accuracy regardless of the position of the initial LS contour is in the image.

### Comparison to U-Net-based deep neural networks

We furthermore compare the segmentation accuracy of the proposed method with those obtained by a deep neural network based on the U-Net architecture^14^ and by one of its descendant variants, BCDU-Net^34^, both of which have been successfully used in several segmentation applications. See supplementary information online and Supplementary Fig. S4 for more details on the networks architectures. The data of 18 subjects are used for training the networks from scratch, 12 subjects are used for the validation, and the data of the remaining 15 subjects are used for testing. To avoid model overfitting, training and validation data are augmented by flipping the images vertically and horizontally, adding Gaussian noise with zero mean and different variances (0.01, 0.02, 0.05) to the already-normalized image intensities, image rotation with different angles ($\pm$ 45°, $\pm$ 90°, 180°), and performing image translation in *x* and *y* direction. The total numbers of images used for training and validation process after applying data augmentation thus become 16,404 and 10,980, respectively. To further increase training data, following^19^, we use the dataset of kidney tumor segmentation (KiTS19) challenge^35^ that includes high quality CT scans for 210 subjects with their ground-truth semantic segmentations. We partition each CT image into two 256 $\times$ 256 sub-images that separately include the left and right kidneys, which further increases the sizes of the training and validation data to 40,050 and 10,980, respectively.

In the training procedure of the two networks, several trials are carried out to tune the model hyper-parameters to ensure the best possible performance on the validation dataset (see the ablation study in the supplementary information online). The models are trained for 200 epochs using Adam optimizer as it is considered the most widely used one among all optimizers^19^. The learning rate is initially set to 0.0001 and is then decayed by a factor of 0.1 whenever the validation loss is not decreased for 10 consecutive epochs. To further avoid overfitting, dropout regularization with 50% ratio is employed during network training. Model training is implemented in a Python environment using Keras APIs with Tensorflow backend and carried out on a workstation with dual 2.20 GHz Intel Xeon Silver 4114 CPUs with 128 GB of RAM and two Nvidia GPUs. After training, the two trained networks are used to segment the kidney from the test images. Table 3 gives a comparison between the results obtained by the proposed FML method versus the U-Net and BCDU-Net results on all images and on the low-contrast images.

**Table 3:** Comparison between the segmentation accuracy of the FML method versus U-Net and BCDU-Net models.

Method	All images		Low-contrast images	
	DSC	HD95	DSC	HD95
U-Net^14^	0.940 ± 0.041	10.30 ± 23.8	0.884 ± 0.071	19.9 ± 28.8
BCDU-Net^34^	0.942 ± 0.038	4.62 ± 12.35	0.90 ± 0.057	7.89 ± 12.27
FML (ours)	**0.961 ± 0.017**	**0.68 ± 1.19**	**0.935 ± 0.037**	**2.23 ± 3.6**

Significant values are in [bold].

**Figure 5:**
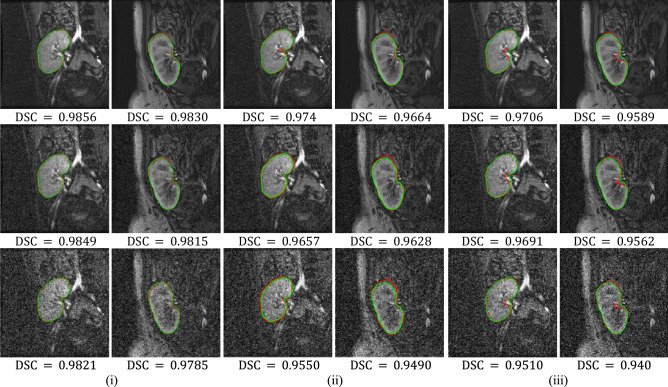
Comparison between the FML method vs U-Net and BCDU-Net models on noisy DCE-MRI images. Segmentation results are shown in red for the FML method in (i), U-Net model in (ii), and BCDU-Net model in (iii). The ground-truth segmentations are shown in green, with DSC reported beneath each result. First row shows original images, second row shows images with noise variance 0.01, and third row shows images with noise variance 0.05.

It is clear from Table 3 that the BCDU-Net model performs notably better than the original U-Net model. However, the FML method performs best on segmenting kidneys from high and low-contrast images. In particular, the mean and standard deviation of HD95 metric reveal that our method is more than 15 × accurate and about 20 × more consistent than the original U-Net model. Moreover, it is about 7 × accurate and 10 × consistent than the BCDU-Net model. To further demonstrate the efficiency of the FML method over the deep models, we apply the three methods to automatically segment the kidney from a set of noisy images. The noisy images are artificially generated by adding Gaussian noise with zero mean and variance values equal 0.01 and 0.05 (note the images are already normalized to range [0, 1]). Figure 5 visually compares between the three methods.

Clearly the performance of the FML method on noisy images is more stable and accurate than those of the U-Net and BCDU-Net models. The improvement is more profound on images with higher noise levels. It is important to mention that the FML method is easier to explain its behavior and interpret its results compared to the deep models. For example, obtaining rather a noisy kidney contour from the segmentation result would suggest increasing the weighting factor ${\uplambda }_{1}$ or ${\uplambda }_{3}$ or both in our method as a corrective action.

## Conclusions

This paper proposes a new method for the automatic and accurate kidney segmentation from DCE-MRI data. It makes the following contributions: (1) It integrates the LS method, FCM clustering, and MRF modeling for this problem *for the first time in the literature*. (2) The FCM clustering algorithm is embedded into the LS method, and the fuzzy memberships of pixels are iteratively updated during the LS contour evolution. This helps to isolate the kidney from the background especially on low-contrast images. (3) The Bayesian parameter estimation method is used to compute the shape prior model of the kidney while accounting for kidney pixels possibly not observed during model building, thus rendering more accurate shape models. The shape model plays an important role in guiding the LS contour evolution. (4) A 2nd-order MGRF model is embedded into the LS formulation to account for the correlation between neighboring pixels.

The FML method has been extensively tested in several experiments on real medical data from 45 subjects. Our experimental results revealed that the FML method can achieve high segmentation accuracy even on noisy or low-contrast images. In all our experiments, the LS contour could converge successfully to the target kidney in the images regardless of where it was initialized and without any tuning of its parameters. The performance of the FML method is found to be better than several state-of-the-art LS methods by more than 0.015 in terms of DSC and 0.63 in terms of HD95. It also offers HD95 improvements of 9.62 and 3.94 over two deep neural networks based on the U-Net model. The improvements are experimentally found to be more profound on low-contrast images as well as DCE-MRI images with high noise levels.

Our current research is directed towards further improving the FML method. One way to do this is to enhance our shape prior model and how it is integrated within our framework. This is because the kidney shape prior information has a key role on guiding the LS contour, and consequently has an important effect on segmentation accuracy especially on low-contrast images. Given that a kidney’s shape would change somewhat from one patient to another, we plan to incorporate a subject-specific shape model together along with a population-based shape model into our LS formulation. Some early work on this new direction has been drafted^36^. We are also working on improving the time performance of the proposed method through converting the MATLAB code to C++ code optimized for GPU computing.

## Supplementary Information

Supplementary Information.

## Data Availability

The datasets used in the current study are available from the authors upon reasonable request and with permission of STDF.

## References

[1] Mostapha M, Khalifa F, Alansary A, Soliman A, Suri J, El-Baz A (2014). Computer-aided diagnosis systems for acute renal transplant rejection: Challenges and methodologies. Abdomen and Thoracic Imaging.

[2] Zöllner FG, Kociński M, Hansen L, Golla AK, Trbalić AŠ, Lundervold A, Materka A, Rogelj P (2021). Kidney segmentation in renal magnetic resonance imaging-current status and prospects. IEEE Access.

[3] Yuksel SE, El-Baz A, Farag A, El-Ghar MA, Eldiasty T, Ghoneim MA (2007). A kidney segmentation framework for dynamic contrast enhanced magnetic resonance imaging. J. Vib. Control.

[4] El-Baz, A. & Gimel’farb, G. Robust medical images segmentation using learned shape and appearance models. In *Proceedings of International Conference on Medical Image Computing and Computer-Assisted Intervention,* 281–288 (London, UK, 2009). 10.1007/978-3-642-04268-3_35 10.1007/978-3-642-04268-3_35 20425998

[5] Khalifa, F., El-Baz, A., Gimel’farb, G. & El-Ghar, M. A. Non-invasive image-based approach for early detection of acute renal rejection. In *Proceedings of International Conference on Medical Image Computing and Computer-Assisted Intervention* 10–18 (2010). 10.1007/978-3-642-15705-9_2 10.1007/978-3-642-15705-9_2 20879209

[6] Khalifa F, Beache GM, El-Ghar MA, El-Diasty T, Gimel'farb G, Kong M, El-Baz A (2013). Dynamic contrast-enhanced MRI-based early detection of acute renal transplant rejection. IEEE Trans. Med. Imaging.

[7] Liu, N., Soliman, A., Gimel’farb, G. & El-Baz, A. Segmenting kidney DCE-MRI using 1st-order shape and 5th-order appearance priors. In *Proceedings of International Conference on Medical Image Computing and Computer-Assisted Intervention* 77–84 (Springer, 2015). 10.1007/978-3-319-24553-9_10

[8] Al-Shamasneh AR, Jalab HA, Palaiahnakote S, Obaidellah UH, Ibrahim RW, El-Melegy MT (2018). A new local fractional entropy-based model for kidney MRI image enhancement. Entropy.

[9] Al-Shamasneh AR, Jalab HA, Shivakumara P, Ibrahim RW, Obaidellah UH (2020). Kidney segmentation in MR images using active contour model driven by fractional-based energy minimization. SIViP.

[10] Hodneland E, Hanson EA, Lundervold A, Modersitzki J, Eikefjord E, Munthe-Kaas AZ (2014). Segmentation-driven image registration-application to 4D DCE-MRI recordings of the moving kidneys. IEEE Trans. Image Process..

[11] Eltanboly A, Ghazal M, Hajjdiab H, Shalaby A, Switala A, Mahmoud A, Sahoo P, El-Azab M, El-Baz A (2019). Level sets-based image segmentation approach using statistical shape priors. Appl. Math. Comput..

[12] Lundervold, A. S., Rørvik, J. & Lundervold, A. Fast semi-supervised segmentation of the kidneys in DCE-MRI using convolutional neural networks and transfer learning. In *Proceedings of 2nd International Scientific Symposium, Functional Renal Imaging: Where Physiology, Nephrology, Radiology and Physics Meet* (Berlin, Germany, 2017).

[13] Haghighi, M., Warfield, S. K. & Kurugol, S. Automatic renal segmentation in DCE-MRI using convolutional neural networks. In *Proceedings of IEEE International Symposium on Biomedical Imaging* 1534–1537 (ISBI, 2018). 10.1109/ISBI.2018.8363865 10.1109/ISBI.2018.8363865 PMC6248325 30473744

[14] Ronneberger, O., Fischer, P. & Brox, T. U-net: Convolutional networks for biomedical image segmentation. In *Proceedings of International Conference on Medical Image Computing and Computer-Assisted Intervention* 234–241 (Springer, 2015). 10.48550/arXiv.1505.04597

[15] Milecki, L., Bodard, S., Correas, J. M., Timsit, M. O. & Vakalopoulou, M. 3D unsupervised kidney graft segmentation based on deep learning and multi-sequence MRI. In *Proceedings of IEEE International Symposium on Biomedical Imaging* 1781–1785. (ISBI, 2021). 10.1109/ISBI48211.2021.9433854.

[16] Bevilacqua V, Brunetti A, Cascarano GD, Guerriero A, Pesce F, Moschetta M, Gesualdo L (2019). A comparison between two semantic deep learning frameworks for the autosomal dominant polycystic kidney disease segmentation based on magnetic resonance images. BMC Med. Inform. Decis. Mak..

[17] Brunetti, A., Cascarano, G. D., Feudis, I. D., Moschetta, M., Gesualdo, L. & Bevilacqua, V. Detection and segmentation of kidneys from magnetic resonance images in patients with autosomal dominant polycystic kidney disease. In *Proceedings of International Conference on Intelligent Computing* 639–650 (Springer, Cham, 2019).

[18] Isensee F, Jaeger PF, Kohl SA, Petersen J, Maier-Hein KH (2021). nnU-Net: A self-configuring method for deep learning-based biomedical image segmentation. Nat. Methods.

[19] Kavur AE, Gezer NS, Barış M, Aslan S, Conze PH, Groza V, Pham DD, Chatterjee S, Ernst P, Özkan S, Baydar B (2021). CHAOS challenge-combined (CT-MR) healthy abdominal organ segmentation. Med. Image Anal..

[20] El-Melegy, M. T., Abd El-karim, R. M., El-Baz, A. & El-Ghar, M. A. Fuzzy membership-driven level set for automatic kidney segmentation from DCE-MRI. In *Proceedings of IEEE International Conference on Fuzzy Systems* 1–8 (FUZZ-IEEE, 20187). 10.1109/FUZZ-IEEE.2018.8491552

[21] Nayak J, Naik B, Behera HS (2015). Fuzzy C-means (FCM) clustering algorithm: A decade review from 2000 to 2014. Comput. Intell. Data Min..

[22] Fedkiw R, Osher S (2002). Level Set Methods and Dynamic Implicit Surfaces.

[23] Lin, D. & Fisher, J. Low level vision via switchable Markov random Fields. In *Proceedings of IEEE International Conference on Computer Vision and Pattern Recognition* 2432–2439 (2012). 10.1109/CVPR.2012.6247957

[24] Friedman, N. & Singer, Y. Efficient Bayesian parameter estimation in large discrete domains. In *Proceedings of the 11 th International Conference on Advances in Neural Information Processing Systems, NIPS'98* 417–423 (1999). http://papers.nips.cc/paper/1616-efficient-bayesian-parameter-estimation-in-large-discrete-domains

[25] El-Melegy M, Mokhtar H (2014). Tumor segmentation in brain MRI using a fuzzy approach with class center priors. EURASIP J. Image Video Process..

[26] Viola P, Wells WM (1997). Alignment by maximization of mutual information. Int. J. Comput. Vis..

[27] Heller, K. A., Svore, K. M., Keromytis, A. D. & Stolfo, S. J. One class support vector machines for detecting anomalous windows registry accesses. 10.7916/D85M6CFF (2003).

[28] Besag J (1974). Spatial interaction and the statistical analysis of lattice systems. J. R. Stat. Soc. Ser. B (Methodol.).

[29] Yang X, Gao X, Tao D, Li X, Li J (2015). An efficient MRF embedded level set method for image segmentation. IEEE Trans. Image Process..

[30] Li Y, Cao G, Wang T, Cui Q, Wang B (2020). A novel local region-based active contour model for image segmentation using Bayes theorem. Inf. Sci..

[31] Tsai A, Yezzi A, Wells W, Tempany C, Tucker D, Fan A, Willsky A (2003). A shape-based approach to the segmentation of medical imagery using level sets. IEEE Trans. Med. Imaging.

[32] El Munim HEA, Farag AA (2007). Curve/surface representation and evolution using vector level sets with application to the shape-based segmentation problem. IEEE Trans. Pattern Anal. Mach. Intell..

[33] Salah MB, Mitiche A, Ayed IB (2010). Multiregion image segmentation by parametric kernel graph cuts. IEEE Trans. Image Process..

[34] Azad, R., Asadi-Aghbolaghi, M., Fathy, M. & Escalera, S. Bi-directional ConvLSTM U-Net with densley connected convolutions.In *Proceedings of the IEEE/CVF International Conference on Computer Vision Workshops* 1–10 (2019). 10.48550/arXiv.1909.00166

[35] Heller, N., Sathianathen, N., Kalapara, A., Walczak, E., Moore, K., Kaluzniak, H., Rosenberg, J., Blake, P., Rengel, Z., Oestreich, M. *et al.* The kits19 challenge data: 300 kidney tumor cases with clinical context, CT semantic segmentations, and surgical outcomes. 10.48550/arXiv.1904.00445 (2019).

[36] El-Melegy, M. T., Abd El-Karim, R. M., El-Baz, A. S. & El-Ghar, M. A. A combined fuzzy C-means and level set method for automatic DCE-MRI kidney segmentation using both population-based and patient-specific shape statistics. In *Proceedings of IEEE International Conference on Fuzzy Systems* 1–8 (FUZZ-IEEE, 2020). 10.1109/FUZZ48607.2020.9177563

