# Supplementary material for: Kidney segmentation from DCE-MRI converging level set methods, fuzzy clustering and Markov random field modeling
Source: Sci Rep. 2022 Nov 5;12:18816. doi: 10.1038/s41598-022-23408-1 (PMC9637091; doi:10.1038/s41598-022-23408-1)
Supplement: Supplementary file 1 — Supplementary Information. [file 41598_2022_23408_MOESM1_ESM.docx]

Supplementary Information

Kidney segmentation from DCE-MRI converging level set methods, fuzzy clustering and Markov random field modeling

# Moumen El-Melegy1,*, Rasha Kamel2, Mohamed Abou El-Ghar3, Mohamed Shehata4, Fahmi Khalifa4,5, and Ayman El-Baz4

1Assiut University, Electrical Engineering Department, Assiut, Egypt

2Assiut University, Computer Science Department, Assiut, Egypt

3Mansoura University, Radiology Department, Urology and Nephrology Center, Mansoura, Egypt

4University of Louisville, Bioengineering Department, Louisville, KY, USA

5Mansoura University, Electronics and Communications Engineering Department, Mansoura, Egypt

Supplementary Table S1. Overview of MRI deep neural network kidney segmentation methods.

| **Reference** | **Method** | **Number of patients**  **Modality** | **DSC** |
| --- | --- | --- | --- |
| Lundervold et al.^12^ | CNNs | 20  DCE-MRIs | 0.87 / 85  Left / Right |
| Haghighi et al.^13^ | Two cascaded 3D U-Nets | 30  Pediatric DCE-MRIs | 0.91 ± 0.03 Normal  0.83 ± 0.03 Abnormal |
| Milecki et al.^15^ | CNNs with thresholding | 32  DCE and T2 MRIs | 0.89 ± 0.0317 |
| Bevilacqua et al.^16^ | CNNs  (VGG-16) | 18  T2-Weighted MRIs | 0.85 |
| Brunetti et al.^17^ | CNNs with  genetic algorithm | 18  T2-Weighted MRIs | 0.91 |
| Isensee et al.^18^ | nnU-Net | 40  T1-DUAL IP/OP MRIs T2-SPIR MRIs | 0.94 ± 0.0159 |

* IP/OP stands for in-phase/oppose-phase, while SPIR stands for spectral pre-saturation inversion recovery.

**Note**: The accuracy reported by Isensee et al.^18^ represents the accuracy regarding the segmentation of all abdominal organs; the specific kidney segmentation performance is not reported.

# Level Set Representation

Supplementary Fig. S1 illustrates the representation of the LS function in the image domain. The level set (LS) function is taken as the signed distance of a pixel from the contour, such that the distance for the pixels inside the contour is positive and negative outside. That is, for every pixel$(x, y)$ in the image, the LS function is defined as follows:

$$\begin{matrix} \phi_{x,y}>0 & if \left( x,y \right)\in\Omega^{K} \\ \phi_{x,y}<0 & if \left( x,y \right)\in\Omega^{B} \\ \phi_{x,y}=0 & if \left( x,y \right)\in\partial\Omega\end{matrix}$$

The interested reader is referred to Reference 22 for more details.

| $\Omega^{K}$  $\phi>0$  $\Omega^{B}$  $\phi<0$  $\partial\Omega$  $\phi=0$ |
| --- |
| Supplementary Figure S1. The representation of a signed distance LS function in the image domain $\boldsymbol{\Omega}$. |

# DCE-MRI Kidney Segmentation using FCM Clustering

The following figure shows the results of the FCM algorithm on segmenting a DCE‑MRI image into two separate clusters. Clearly, depending *only* on fuzzy membership clusters is not effective and fails to generate accurate results.

|  | 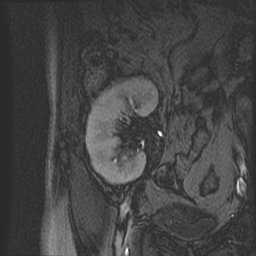 | 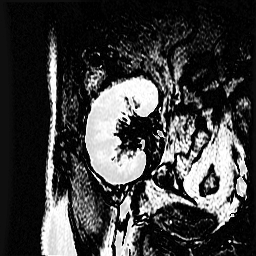 | 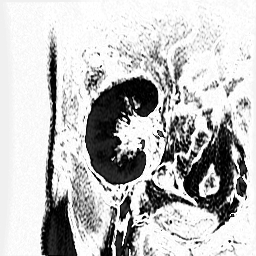 |  |
| --- | --- | --- | --- | --- |
|  | (a) | (b) | (c) |  |
| **Supplementary Figure S2.** DCE-MRI kidney segmentation using FCM clustering algorithm. (a) Input image, (b) Kidney cluster, and (c) Background cluster. | | | | |

# Algorithms of the Proposed Method

Algorithm S1 explains the steps of constructing the shape prior information model, which is done offline from a set of ground-truth kidneys of different subjects. Algorithm S2 explains the steps of the proposed FML kidney segmentation method, which is run on the subject’s sequence to be segmented.

| Algorithm S1. Kidney shape model construction |
| --- |
| Input: A set of DCE-MRI images of different subjects and their binary ground-truth segmentations.  Output: Constructed shape model.   1. Pick one image among the DCE-MRI images as a reference image. 2. Assuming affine transformation, register all images in the set to the selected reference image using mutual information maximization^26^. 3. Build the shape model from the registered ground‑truth kidney segmentations using the Bayesian parameter estimation method as explained in Section “Kidney shape prior model”. |

| Algorithm S2. FML algorithm for DCE-MRI kidney segmentation |
| --- |
| Input: One subject’s sequence to be segmented.  Output: A sequence of binary segmented kidneys.   - For each image in the sequence do the following:  1. Align the input image to the reference image selected in shape model construction assuming affine transformation. 2. Equalize the image using its cumulative histogram. 3. Initialize the contour (e.g., near image borders or even randomly) and initialize the LS function $\phi$. 4. Initialize the centroid of kidney and background clusters as the average of pixel intensities inside and outside the initialized LS contour. 5. Generate initial kidney and background memberships using the centroid values according to (9). 6. Compute mean and standard deviation of pixel intensities in kidney and background regions using (15). 7. Compute kidney and background MRF energy functions for each pixel using (18). 8. Evolve the LS contour according to (6) and (7). 9. Update centroid values and fuzzy membership clusters according to (9) and (10). 10. Repeat steps 6 - 9 until the energy functional (1) is stable or a maximum number of repetitions has been reached. |

# Sample Segmentation Results

Supplementary Fig. S3 shows the segmentation results of the proposed level set-based method with its best performing parameter values. The first row depicts a number of DCE-MRI kidney images of different subject with the initial contour initializations outlined in red. The second row shows the kidneys segmented by the FML method in red with their DSC values. The ground-truth segmentations are outlined in green in the third row.

|  | 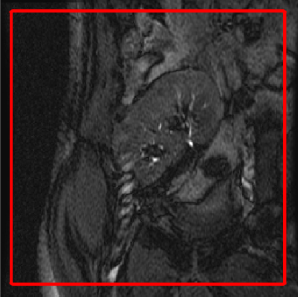 | 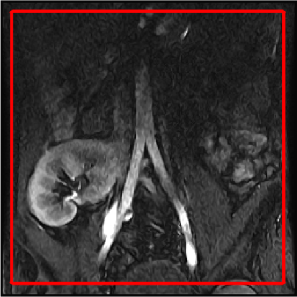 | 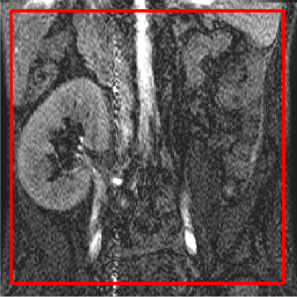 | 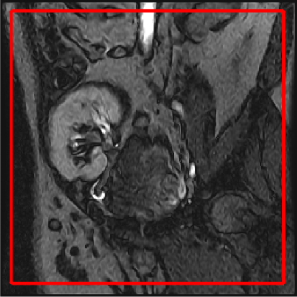 | 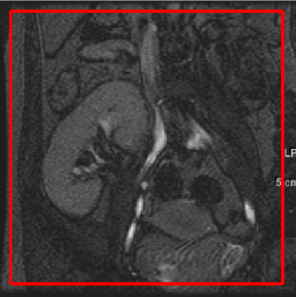 |  |
| --- | --- | --- | --- | --- | --- | --- |
|  | $t=1$ | $t=4$ | $t=23$ | $t=60$ | $t=72$ |  |
|  | 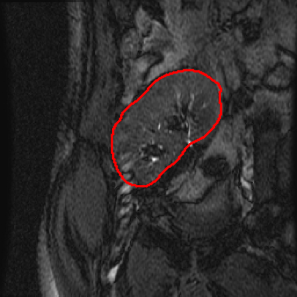 | 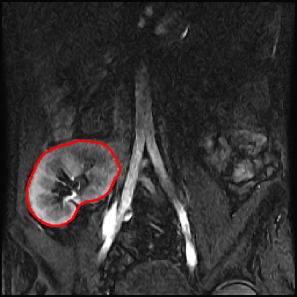 | 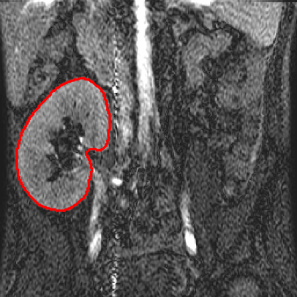 | 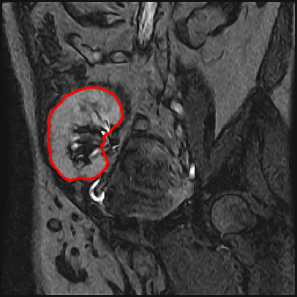 | 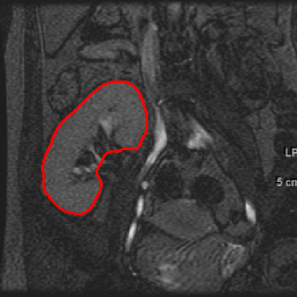 |  |
|  | $DSC = 0.9534$ | $DSC = 0.976$ | $DSC = 0.974$ | $DSC = 0.956$ | $DSC = 0.956$ |  |
|  | 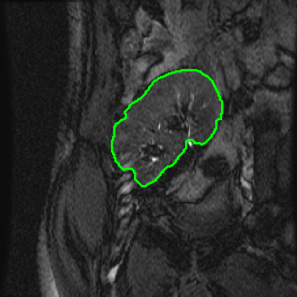 | 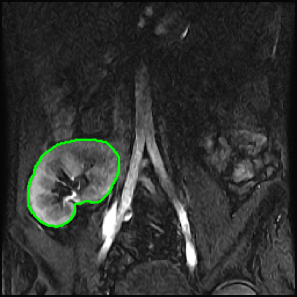 | 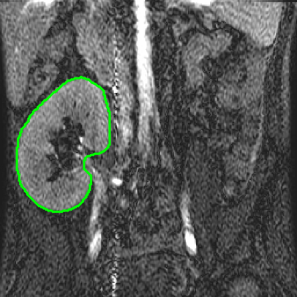 | 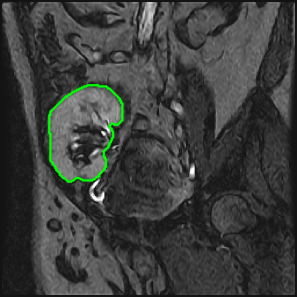 | 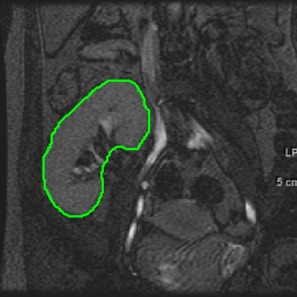 |  |
| Supplementary Figure S3. Segmentation results of our FML method. First row shows DCE-MRI kidney images with initial LS contour. Second row shows segmented kidneys in red with their DSC values. Third row shows ground-truth segmentations in green. | | | | | | |

# Parameter Analysis Experiments for the Proposed Level set-based Method

We here perform several experiments to investigate the influence of changing various parameters on the overall performance of the proposed method. For all experiments, we use the DSC and HD95 metrics for assessing the segmentation performance on all test images as well as on the low-contrast images of the test set. First, we carry out experiments to study the impact of changing the values of $\lambda_{2}$ and $\lambda_{3}$ that control the contributions of FCM and MRF energy terms on the method’s performance. We then examine the proposed method’s performance against the neighborhood size $w$ values. Afterwards, we evaluate the performance of our level set method with different Gibbsian parameter $\gamma$ values. In all experiments, the smoothness parameter $\lambda_{1}$ in equation (6), the width of numerical smearing $\varepsilon$ in equation (3), and pseudo count $\beta$ in equation (11) are set and fixed to 6, 1.5, and 1, respectively. Quantitative results are shown in Supplementary Table S2, where the bold values denote the best result.

Note that the first table row represents the performance when the MRF-based component of the evolution equation (6) governing the level set iterations is cancelled. Clearly this component has a positive impact on the performance as evident from the comparison with the best results in the 4^th^ row where both the FCM and MRF components are considered and weighted equally.

Supplementary Table S2. Segmentation performance of the proposed method with various parameter values.

| **Experiment** | $\boldsymbol{\lambda}_{\boldsymbol{2}}\boldsymbol{:}\boldsymbol{\lambda}_{\boldsymbol{3}}$ | $\boldsymbol{w}$ | $\boldsymbol{\gamma}$ | **All Images** | | **Low-contrast Images** | |
| --- | --- | --- | --- | --- | --- | --- | --- |
|  |  |  |  | **DSC** | **HD95** | **DSC** | **HD95** |
| 1 | 6 : 0 | 5 | 0.5 | 0.946 ± 0.029 | 1.63 ± 1.97 | 0.918 ± 0.06 | 3.18 ± 4.28 |
| 2 | 6 : 1 | 5 | 0.5 | 0.945 ± 0.040 | 1.62 ± 2.25 | 0.90 ± 0.048 | 2.65 ± 2.34 |
| 3 | 6 : 3 | 5 | 0.5 | 0.949 ± 0.036 | 1.48 ± 1.66 | 0.928 ± 0.029 | 2.25 ± 2.03 |
| 4 | 6 : 6 | 5 | 0.5 | **0.956 ± 0.019** | **1.15 ± 1.46** | **0.936 ± 0.024** | **1.94 ± 1.58** |
| 5 | 6 : 8 | 5 | 0.5 | 0.942 ± 0.044 | 1.85 ± 2.08 | 0.93 ± 0.026 | 2.31 ± 2.06 |
| 6 | 6 : 6 | 5 | 0.1 | 0.946 ± 0.042 | 1.74 ± 2.0 | 0.934 ± 0.027 | 2.28 ± 2.09 |
| 7 | 6 : 6 | 5 | 0.9 | 0.944 ± 0.041 | 1.8 ± 1.98 | 0.93 ± 0.026 | 2.45 ± 2.25 |
| 8 | 6 : 6 | 7 | 0.5 | 0.950 ± 0.035 | 1.4 ± 1.7 | 0.930 ± 0.028 | 2.4 ± 2.37 |
| 9 | 6 : 6 | 9 | 0.5 | 0.949 ± 0.036 | 1.53 ± 1.82 | 0.920 ± 0.031 | 2.6 ± 2.03 |

# U-Net and BCDU-Net Deep Neural Networks

Deep neural networks based on U‑Net architecture^14^ and its variations have been successfully used in many segmentation applications. In this work, we investigate the original U-Net model and one of its variants named BCDU-Net model for DCE-MRI kidney segmentation. The original U-Net model typically consists of two parts: the left side serving as a contracting path and the right side as an expansive path, as shown in Supplementary Fig. S4. Each layer in contracting path contains two 3×3 convolutional layers followed by a dropout layer to prevent overfitting, a rectified linear unit (ReLU) activation function which sets all negative outputs to 0, and a 2×2 max-pooling layer that doubles the number of the feature channels and halves the size of the image. Conversely, each decoder layer has a 2×2 up-convolution operation that halves the number of feature channels. Each up-convolved feature map is concatenated with the corresponding feature map from the contracting path. The model ends with a 1×1 convolutional layer that uses a sigmoid activation function and produces feature maps of the same size as the input image.

On the other hand, BCDU-Net^34^ model inherits the advantages of U-Net^14^, bidirectional convolutional long short term memory (BConvLSTM), and dense convolutions. Each layer in the contracting path of BCDU-Net model consists of two 3×3 convolutional filters followed by a ReLU activation function, dropout layer, and 2×2 max-pooling layer. In contrast to U-Net model, the last convolutional layer of encoding path in BDCU-Net model includes a sequence of densely connected convolutions, in which, feature maps of all previous layers are concatenated with feature map of current layer and used as input for the next convolution. Each layer in the decoding path starts by executing a 2×2 up-sampling operation over the previous layer’s output followed by batch normalization function. The feature maps resulting from up-convolution operation are combined with the corresponding feature maps of the contracting path employing BConvLSTM. As in the U-Net model, a sigmoid activation function is used at the end of the model. In our experiments, we use BCDU-Net model with three dense blocks.

Supplementary Fig. S4 illustrates the architectures of both deep networks.

| 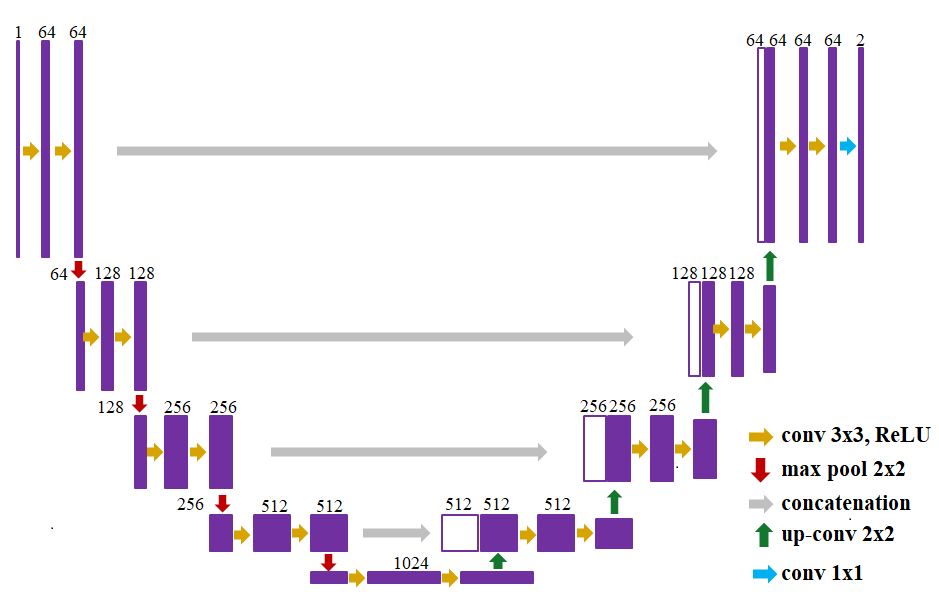 |
| --- |
| 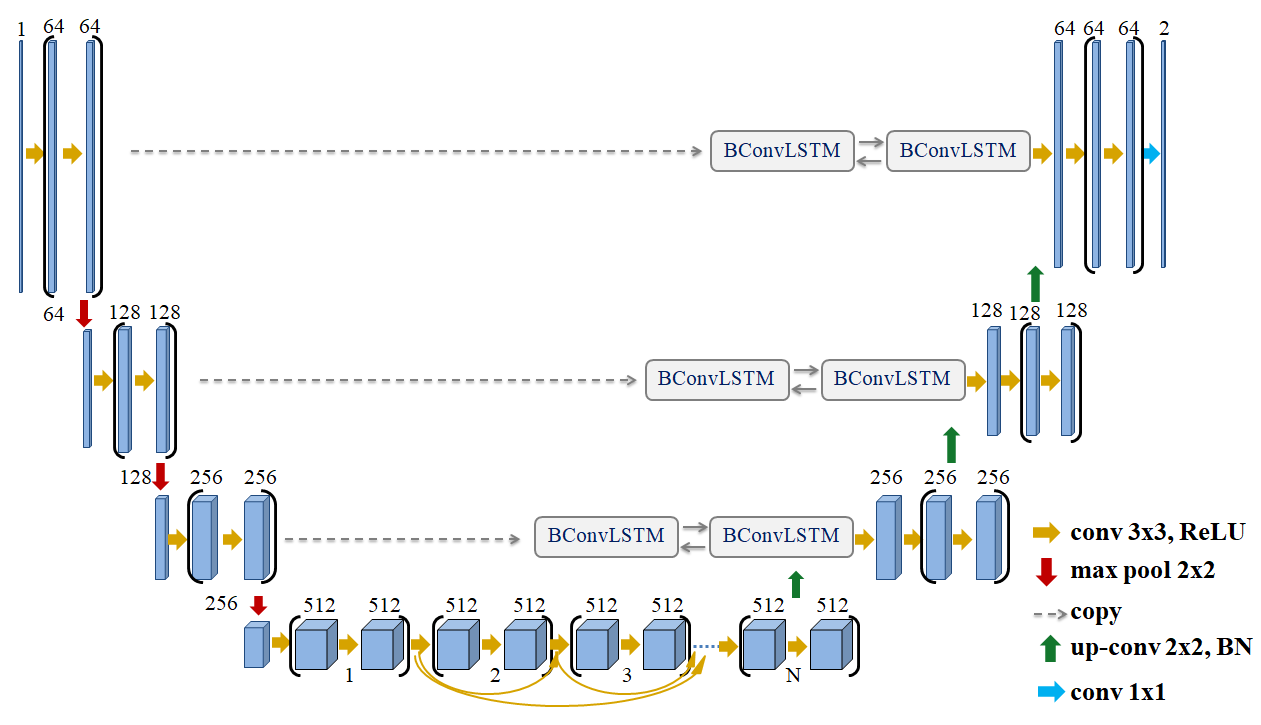 |
| Supplementary Figure S4. Architecture of original U-Net model (top) and BCDU-Net model (bottom). |

# Ablation Experiments for Deep Neural Network Model

The BCDU-Net model has outperformed the original U-Net for DEC-MRI kidney segmentation in our experiments. Thus, we perform several experiments to further investigate the BCDU-Net model’s performance with various hyper-parameters. In this study, we choose the most important parameters to explore their impacts on the performance. For all experiments, we use the DSC and HD95 metrics for assessing the segmentation performance on all test images as well as on the low-contrast images of the test set. We first test the model with two of the most common used loss functions, namely, binary cross entropy (BCE) and summation of DSC and BCE (DSC-BCE). Afterwards, we explore the impact of changing the dropout regularization values on the segmentation performance. Additionally, we analyze the model’s behavior with different initial learning rates. In all experiments, the learning rate is set to decay by a factor of 0.1 when validation loss is not decreased for 10 consecutive epochs. Quantitative results are shown in Supplementary Table S3, where the bold values denote the best result.

Supplementary Table S3. Segmentation performance of BCDU-Net model with different hyper-parameters values.

| **Experiment** | **Loss Function** | **Initial Learning Rate** | **Dropout** | **All Images** | | **Low-contrast Images** | |
| --- | --- | --- | --- | --- | --- | --- | --- |
|  |  |  |  | **DSC** | **HD95** | **DSC** | **HD95** |
| 1 | BCE | 0.0001 | 0.1 | 0.929 ± 0.11 | 5.77 ± 16.85 | 0.72 ± 0.28 | 26.9 ± 32.9 |
| 2 | BCE | **0.0001** | **0.5** | **0.942 ± 0.038** | **4.62 ± 12.35** | **0.90 ± 0.057** | **7.89 ± 12.27** |
| 3 | BCE | 0.0001 | 0.8 | 0.946 ± 0.046 | 8.574 ± 21.15 | 0.915 ± 0.056 | 13.43 ± 23.56 |
| 4 | DSC-BCE | 0.0001 | 0.5 | 0.94 ± 0.053 | 7.24 ± 17.87 | 0.88 ± 0.13 | 12.2 ± 20.6 |
| 5 | BCE | 0.001 | 0.5 | 0.92 ± 0.068 | 16.27 ± 25.47 | 0.81 ± 0.15 | 26.9 ± 29.42 |
| 6 | BCE | 0.0005 | 0.5 | 0.90 ± 0.25 | 25.57 ± 31.67 | 0.87 ± 0.26 | 49.09 ± 31.8 |
